# Blood Product Transfusion and Antifibrinolytic Choice Are Not Associated with Higher Rates of Intraluminal Thrombus Following Non-Emergency Aortic Arch Replacement Surgery

**DOI:** 10.3390/jcm15155886

**Published:** 2026-07-28

**Authors:** Rizwan Barkat, Ben Chisnall, Justin Fong, Daniel Aston, Florian Falter

**Affiliations:** 1 Our Lady of Lourdes Hospital, A92 VW28 Drogheda, Ireland; rizwan.barkat@hse.ie; 2 Royal Papworth Hospital, Cambridge CB2 0AY, UK; ben.chisnall@nhs.net (B.C.); justin.fong@health.nsw.gov.au (J.F.); daniel.aston@nhs.net (D.A.); 3 St. George Hospital, Sydney, NSW 2217, Australia

**Keywords:** frozen elephant trunk, intraluminal thrombus, aortic arch replacement, blood transfusion, patient blood management, coagulopathy, antifibrinolytics

## Abstract

**Background**: Intraluminal thrombus (ILT) is a recognized complication of aortic arch replacement surgery using a frozen elephant trunk (FET) prosthesis, with a postoperative incidence of around 10%. Risk factors for ILT include aneurysmal aortic disease, central graft positioning, and prothrombotic medical conditions. Perioperative blood product transfusion is frequently required in these patients to correct postoperative coagulopathy following cardiopulmonary bypass (CPB). We hypothesized that there may be an association between (i) intra- and postoperative blood product transfusion volumes and the development of ILT and (ii) whether the use of either aprotinin or tranexamic acid is associated with postoperative ILT formation. **Methods**: Retrospective analysis was carried out on all non-emergency aortic arch replacement procedures at our institution with FET implantation between January 2017 and June 2025. ILT was defined as the presence of thrombus within the aortic graft identified on postoperative computed tomography (CT). Statistical analysis was performed on transfusion data from anesthesia and intensive care unit records. Secondary analysis compared ILT incidence and blood product transfusion volume between patients receiving aprotinin versus tranexamic acid as antifibrinolytic therapy. **Results**: Of 162 patients, 10 (6.2%) developed postoperative ILT. All patients received blood product transfusion either intraoperatively or postoperatively. There was no statistically significant difference in the volume of individual blood products (red cells, platelets, fresh frozen plasma, cryoprecipitate, or fibrinogen concentrate) received between the ILT and non-ILT groups. The mean total blood product transfusion was 13.50 units (ILT group) versus 11.10 units (no ILT group), with *p* = 0.897, indicating no significant association. Among 46 patients receiving aprotinin, only one (2.2%) developed ILT, whereas nine of 114 patients (7.8%) receiving tranexamic acid developed ILT. This difference was not statistically significant (*p* = 0.232). **Conclusions**: In this single-centre retrospective cohort study, no statistically significant association was detected between perioperative blood product transfusion volume or choice of antifibrinolytic agent (aprotinin versus tranexamic acid) and intraluminal thrombus formation following non-emergency aortic arch replacement surgery. However, the small number of ILT events (n = 10) limits statistical power, and a clinically meaningful association cannot be excluded. These findings provide preliminary reassurance that correction of intra- and postoperative coagulopathy may not increase ILT risk, though larger multicentre studies with adjustment for confounding variables including postoperative anticoagulation practices are warranted.

## 1. Introduction

Complex aortic arch surgery remains one of the most challenging procedures in cardiothoracic surgical practice. The frozen elephant trunk (FET) technique, which combines total aortic arch replacement with antegrade endovascular repair of the proximal descending aorta, has become the standard approach for managing extensive aortic pathologies involving the arch and descending thoracic aorta [1,2]. This hybrid technique offers the theoretical advantage of addressing the proximal pathology whilst facilitating future endovascular completion, reducing the need for extensive open thoracoabdominal aortic repair.

Despite the technical advantages of the FET procedure, significant challenges persist with perioperative management. Aortic arch surgery frequently involves prolonged cardiopulmonary bypass times, deep hypothermic circulatory arrest, and selective antegrade cerebral perfusion, all of which are associated with profound coagulopathy and substantial blood loss [3]. The resulting coagulopathy necessitates aggressive transfusion of blood products intra- and postoperatively, raising concerns about potential complications including thrombotic events.

One recognized complication specific to FET procedures is intraluminal thrombus (ILT) formation within the stent graft component. The incidence of ILT after FET has been reported as between 6% and 17% in various series, with associated increased morbidity and mortality [4,5]. Multiple risk factors for ILT development have been identified, including aneurysmal aortic disease, central graft positioning, and prothrombotic medical conditions. The phenomenon of thrombus formation within the graft lumen remains incompletely understood, with several mechanistic theories proposed. Anatomical factors such as slow or turbulent flow, particularly with larger stent grafts or undersizing, appear to play a significant role [6].

An important clinical question remains unresolved: does perioperative blood product transfusion, necessary for correcting coagulopathy and managing bleeding, contribute to ILT formation? While one hypothesis suggests that aggressive transfusion strategies may promote a prothrombotic state [7], the relationship between transfusion volume and ILT development has not been systematically studied. Currently, there is a knowledge gap regarding whether transfusion practices independently influence ILT risk or whether anatomical and haemodynamic factors are the predominant determinants.

At our institution, we apply comprehensive patient blood management protocols utilizing viscoelastic testing (ROTEM; Werfen, Barcelona, Spain) to guide transfusion decisions [8]. This approach aims to correct identified coagulation defects whilst minimizing unnecessary transfusion. To investigate whether our transfusion strategies contribute to ILT development, we undertook a retrospective analysis comparing blood product administration in patients who did and did not develop postoperative ILT following non-emergency aortic arch replacement with FET.

Antifibrinolytics, such as the lysine analogue tranexamic acid (TXA) and serin protease inhibitor aprotinin, are regularly used during cardiac surgery and have been shown to reduce transfusion requirements by up to 50% by inhibiting plasmin activity [9]. While guidance by the UK National Institute for Health and Care Excellence (NICE) all but mandates the use of TXA in procedures associated with moderate or more blood loss, the use of aprotinin is more controversial [10]. In our institution it is used on a named patient basis in accordance with team preferences. Alongside the primary focus on blood product transfusion and ILT formation, this retrospective analysis investigates whether there is a difference in rates of ILT development and/or transfusion volume between patients who received TXA and patients who received aprotinin.

## 2. Materials and Methods

### 2.1. Study Design and Patient Selection

This was a single-centre retrospective cohort study of all consecutive patients who underwent non-emergency total aortic arch replacement with frozen elephant trunk implantation at our institution between January 2017 and June 2025. The study was reviewed and approved by the institutional review board, which waived the requirement for informed consent given the retrospective nature (Reference Non-HRA0025). Non-emergency procedures were defined as elective cases or those undergoing planned delayed surgery following initial medical management of aortic disease. A total of 162 patients met the inclusion criteria. We excluded patients with acute aortic syndromes (acute aortic dissection or acute intramural hematoma) requiring emergency intervention, as the pathophysiology and transfusion requirements differ significantly from elective cases. Patient identification was performed using institutional operative records and electronic medical record systems.

### 2.2. Clinical Data Collection

Comprehensive demographic, operative, and transfusion data were extracted from multiple sources: the institutional surgical database (CARDS II), the electronic medical record (Lorenzo, Daedalus, Florence, Italy), intraoperative anesthetic records (Metavision version 5.46.44, IMDsoft, Tel Aviv, Israel), and the transfusion laboratory database. Data collected (where available) included:

- Demographic variables: age, sex, body mass index, selected comorbidities
- Operative details: operative cardiopulmonary bypass time, aortic cross-clamp time, duration, time of procedure, antifibrinolytic use (aprotinin or TXA)
- Transfusion data: all units of red blood cells, fresh frozen plasma, platelets, cryoprecipitate, fibrinogen concentrate administered from the time of anesthetic induction through 48 h postoperatively
- Postoperative outcomes: chest drain output at 6, 24, and 48 h; postoperative mortality; complications including renal dysfunction, neurological events, and re-exploration for bleeding.

### 2.3. Intraluminal Thrombus Identification

**Figure 1:**
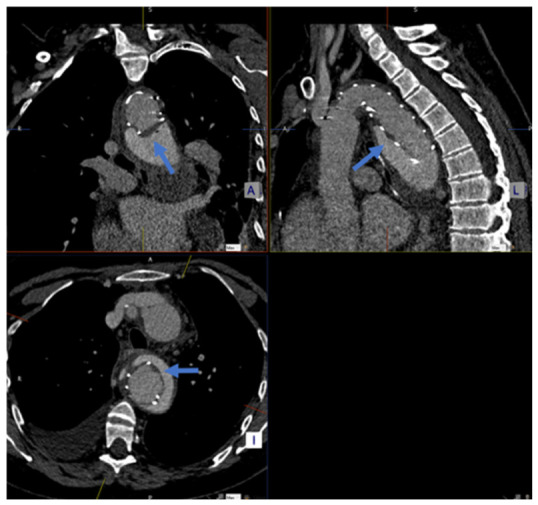
Computed tomography angiography (CTA) demonstrating intraluminal thrombus (ILT) within a frozen elephant trunk graft. This representative axial CTA image shows the characteristic appearance of intraluminal thrombus formation within the stent graft component of a frozen elephant trunk prosthesis (ILT shown as hypodense/dark area indicated by blue arrow).

Intraluminal thrombus was defined as the presence of visible thrombus within the lumen of the aortic graft prosthesis identified on postoperative computed tomography angiography (CTA) imaging at any point during the follow-up period (see Figure 1 for an example of ILT). All CTA scans were performed using standardized protocols at our institution, with multiplanar reformats reviewed to assess for thrombus. CTA imaging was reviewed by consultant radiologists who had no insight into intraoperative transfusion management and were not involved in intraoperative decision-making. All 162 patients underwent at least one postoperative CTA scan. Imaging was routinely performed within the first six to twelve months postoperatively, with additional imaging obtained if clinically indicated by changes in haemodynamic status or surrogate markers of complications.

### 2.4. Transfusion Protocol

In accordance with national guidelines and evidence-based practice, all cardiac surgical patients at our institution receive prophylactic antifibrinolytic therapy intraoperatively [11]. The specific agent (tranexamic acid or aprotinin) and dosing are determined at the discretion of the consultant surgeon and anesthetist. The choice of antifibrinolytic agent was influenced by surgeon and anaesthetist preferences, perceived bleeding risk, procedural complexity (including redo cardiac surgery), and patient-specific factors. Aprotinin was typically reserved for patients considered at higher risk of bleeding or those undergoing redo procedures. Red blood cell transfusion targets an intraoperative hemoglobin of >70 g/L. Administration of coagulation factors and blood products beyond red cells is guided by viscoelastic testing using ROTEM analysis, with defined thresholds guiding therapeutic interventions rather than prophylactic transfusions. Specifically, fibrinogen concentrate or cryoprecipitate was administered when FIBTEM A5 was <9 mm; platelet transfusion was triggered by EXTEM A5 < 50 mm with adequate fibrinogen (FIBTEM A5 ≥ 9 mm); and fresh frozen plasma was given when EXTEM CT was >90 s despite adequate fibrinogen levels.

### 2.5. Statistical Analysis

Demographic and clinical characteristics are presented as mean ± standard deviation, median with interquartile ranges, or count with percentages as appropriate. All statistical comparisons between the ILT and non-ILT groups were performed using the non-parametric Mann–Whitney U testing given the small sample size in the ILT group and non-normal distribution of transfusion data.

Categorical variables were compared using Chi-squared or Fisher’s exact tests. A significance level of *p* < 0.05 was used for all comparisons. Statistical analysis was performed using IBM SPSS version 27 (Armonk, NY, USA) and R software (version 4.5.3, R-Foundation, Vienna, Austria).

## 3. Results

### 3.1. Patient Cohort and Incidence of Intraluminal Thrombus

Of 162 patients who underwent non-emergency aortic arch replacement with FET, 10 patients (6.2%) developed radiographically confirmed intraluminal thrombus. This incidence is consistent with published series reporting ILT rates between 6 and 17% [4,5]. The remaining 152 patients (93.8%) had no evidence of ILT on follow-up imaging.

### 3.2. Demographic and Operative Characteristics

The ILT and non-ILT groups were well-matched with respect to demographic parameters. The mean age was 68.6 years (ILT group) versus 68.47 years (no ILT group). Male predominance was observed in both groups, with 40.0% male in the ILT group and 59.9% in the non-ILT group. Operative parameters including cumulative cardiopulmonary bypass time (252.7 vs. 245.6 min) and aortic cross-clamp time (102.5 vs. 103.4 min) were comparable between groups. There was no statistically significant difference in postoperative chest drain output between the groups (see Table 1 for full descriptive variables).

### 3.3. Blood Product Transfusion Analysis

A detailed comparison of blood product administration is presented in Table 2. There was no statistically significant difference in the volume of any blood product administered between the ILT and non-ILT groups.

The lack of statistically significant differences across all categories of blood products suggests that transfusion strategies were not a determinant of ILT formation in this cohort. Although the ILT group received slightly higher volumes of red blood cells, FFP, and fibrinogen concentrate, these differences did not reach statistical significance. However, given the small number of ILT events (n = 10), this study may lack adequate statistical power to detect clinically meaningful differences, and a type II error cannot be excluded.

### 3.4. Antifibrinolytic Use and Ilt Development

To examine whether specific antifibrinolytic choices influenced ILT rates, we analyzed the relationship between aprotinin and tranexamic acid use and ILT occurrence. Among 46 patients receiving aprotinin, only one (2.2%) developed ILT, whereas nine of 114 patients (7.8%) receiving tranexamic acid developed ILT. This difference was not statistically significant (*p* = 0.232). However, these findings should be interpreted with caution due to the non-randomized allocation of antifibrinolytic agents. Aprotinin was preferentially administered to patients perceived as higher risk or undergoing redo procedures, introducing potential selection bias that may confound the comparison. The absence of a statistically significant difference does not exclude the possibility of a true effect, and a type II error remains possible given the limited number of ILT events.

### 3.5. Antifibrinolytic Use and Blood Product Transfusion

The mean blood product volumes transfused, split into patients who received aprotinin and those who received tranexamic acid as antifibrinolytic therapy, is shown in Table 3. There were statistically significant differences in blood products received for all blood products combined (aprotinin 13.33 vs. TXA 10.45, *p* = 0.003); FFP (4.46 vs. 3.35 units, *p* <0.001); and platelets (2.17 vs. 1.88, *p* = 0.028). There was no significant difference in the volume of red cells, cryoprecipitate, or fibrinogen concentrate transfused depending on whether patients received TXA or aprotinin.

## 4. Discussion

This retrospective analysis of 162 patients undergoing non-emergency aortic arch replacement with FET implantation represents the first study to systematically examine the relationship between perioperative blood product transfusion and subsequent intraluminal thrombus formation. Our principal finding—that no statistically significant association was detected between transfusion volume and ILT development—may have implications for patient blood management in this high-risk population, though interpretation is limited by the small number of ILT events.

### 4.1. Clinical Significance of Findings

The absence of a statistically significant association between transfusion requirements and ILT formation suggests that mechanisms underlying thrombus development in FET grafts may be dominated by anatomical and haemodynamic factors rather than transfusion-related factors. This interpretation is supported by prior mechanistic studies demonstrating that oversizing, undersizing, graft position, and slow flow characteristics are primary determinants of intraluminal thrombus risk [6,12]. Our findings provide preliminary reassurance to clinicians that rational, goal-directed transfusion strategies guided by viscoelastic testing may not substantially increase ILT risk, though the study’s power limitations preclude definitive conclusions. Our data suggest that blood product administration, when guided by correction of identified coagulopathy defects and specified target hemoglobin levels, remains an appropriate therapeutic intervention for managing perioperative bleeding.

### 4.2. Alternative Risk Factors for Intraluminal Thrombus

Recent research has identified several patient and procedural variables associated with ILT development that may be more clinically significant than transfusion volume. Prothrombotic medical conditions—including malignancy, thrombophilia, and antiphospholipid syndrome—have been associated with ILT [5]. Additionally, specific anatomical factors appear critical: central graft positioning (versus eccentric), smaller stent graft diameter-to-aorta ratios, and larger proximal aortic sizes have all been linked to ILT occurrence [13,14]. A recent comprehensive meta-analysis demonstrated that female sex, older age, aortic aneurysm (as opposed to dissection), and embolic events were significantly associated with ILT [3].

The present data do not permit definitive identification of ILT risk factors in our cohort, as the small number of ILT cases (n = 10) limits statistical power for multivariate analyses. Furthermore, our study did not systematically collect data on graft characteristics (size, positioning) or indication for surgery (aneurysm versus dissection), which may confound our findings. Although the demographic and operative characteristics appeared comparable between ILT and non-ILT groups, unmeasured confounding variables—including graft-specific factors and prothrombotic conditions—may have influenced the results.

### 4.3. Management of Coagulopathy in Aortic Arch Surgery

Cardiopulmonary bypass induces complex alterations in haemostasis through multiple mechanisms: haemodilution, platelet dysfunction, consumption and inactivation of coagulation factors, hypothermia-related impairment of enzymatic reactions, and activation of fibrinolysis [15]. The magnitude of these abnormalities often necessitates perioperative transfusion to restore haemostatic capacity and maintain hemoglobin levels adequate for oxygen delivery.

The use of ROTEM-guided transfusion algorithms at our institution permits identification of specific coagulation defects with targeted correction using appropriate blood products or haemostatic agents [8,16]. Evidence from multiple centres demonstrates that viscoelastic-guided algorithms reduce transfusion requirements whilst maintaining effective clotting [17]. This approach is consistent with contemporary patient blood management principles emphasizing individualized, goal-directed strategies rather than prophylactic transfusions [11].

### 4.4. Association of Antifibrinolytic with Transfusion Volume

In our subgroup analysis of whether patients received aprotinin or TXA, there was no significant difference in ILT formation, but a significant difference was evident in the volume of perioperative transfusion. Patients receiving aprotinin received significantly higher volumes of FFP and platelets, which translated into a significantly higher overall perioperative transfusion volume. As we did not have a standardized protocol for whether TXA or aprotinin was chosen for antifibrinolysis, the explanation for this may be that aprotinin was chosen for patients who were felt to be higher risk or were undergoing redo cardiac surgical procedures. Previous studies have examined aprotinin versus tranexamic acid for effects on postoperative mortality, bleeding risk, renal failure, and the need for re-exploration [17,18,19]. An association between aprotinin and reduced blood product transfusion has been shown for coronary artery bypass grafting (CABG) and aortic valve replacement (AVR) surgery [20], and a small study has shown reduced blood product use with aprotinin in patients undergoing thoracic aortic surgery with deep hypothermic circulatory arrest [21]. None of these studies have looked at a larger cohort of patients undergoing major aortic procedures, as our study has. Our findings warrant further exploration in a larger study with adjustment for additional risk factors such as more complex procedural considerations, with the most pertinent of these being whether it is redo aortic surgery.

### 4.5. Postoperative Anticoagulation Strategies

A critical unmeasured variable in our analysis is postoperative anticoagulation practice, which may represent the most important modifiable factor influencing ILT occurrence. Prior studies have demonstrated that early introduction of anticoagulation—particularly with warfarin or direct oral anticoagulants—significantly reduces ILT incidence [4]. The protective effect of anticoagulation suggests that ILT is fundamentally a thrombotic phenomenon driven by intrinsic properties of blood stasis and endothelial injury rather than transfusion-induced procoagulability.

### 4.6. Limitations

This study carries several important limitations that warrant careful consideration. First, as a single-centre retrospective analysis, generalizability may be limited to institutions with similar patient populations, operative techniques, and transfusion protocols. The small number of ILT cases (n = 10) substantially limits statistical power to detect clinically meaningful associations, and a type II error cannot be excluded. This limitation prevents meaningful multivariable analysis of potential confounding variables. We were unable to precisely quantify the timing of transfusions (intraoperative versus postoperative), which might reveal critical windows of vulnerability.

Second, the study is observational and cannot definitively establish causation. Although no statistically significant association was detected between transfusion and ILT, unmeasured confounding variables—such as graft characteristics, indication for surgery (aneurysm versus dissection), redo surgery status, specific intraoperative haemodynamic parameters, adequacy of anticoagulation during CPB, or genetic predisposition to thrombosis—might independently influence both transfusion requirements and ILT risk.

Third, and critically, we did not systematically capture postoperative anticoagulation strategies, which prior evidence suggests may be the most important modifiable factor influencing ILT occurrence. The absence of this variable represents a significant limitation, as postoperative anticoagulation may confound any potential relationship between transfusion and ILT. Future prospective studies incorporating comprehensive anticoagulation data would substantially strengthen conclusions.

Fourth, all patients in this study received either tranexamic acid or aprotinin as antifibrinolytic therapy; no control group without antifibrinolytic agents was available as this would be unethical and contrary to current clinical guidelines for cardiac surgical practice. The non-randomized allocation of aprotinin versus tranexamic acid, based on clinician preference and perceived patient risk, introduces potential selection bias that may confound the comparison between these agents.

### 4.7. Clinical Implications and Future Directions

Our findings have potential implications for perioperative management, though the small sample size and observational design warrant cautious interpretation. The absence of a statistically significant association between transfusion volume and ILT suggests that rational, goal-directed transfusion strategies may not substantially increase ILT risk. Correction of coagulopathy through appropriate blood product administration appears to remain necessary and appropriate in this high-risk population. This supports the continued use of patient blood management principles and viscoelastic-guided transfusion algorithms. To advance the field, future multicentre prospective studies should: (1) collect systematic data on blood product transfusion with precise timing information, (2) incorporate a comprehensive coagulation assessment including viscoelastic parameters and biomarkers of fibrinolysis, (3) standardize and record postoperative anticoagulation protocols and document adherence, (4) perform detailed anatomical analysis of graft characteristics and positioning, (5) systematically record whether procedures are primary or redo operations, and (6) potentially include additional risk factor screening for thrombophilia in ILT cases. Such data would permit identification of modifiable risks for ILT and inform evidence-based prevention strategies.

## 5. Conclusions

This single-centre retrospective study of 162 patients undergoing non-emergency aortic arch replacement with frozen elephant trunk implantation found no statistically significant association between the volume of perioperative blood product transfusion or choice of antifibrinolytic agent and intraluminal thrombus formation. However, the small number of ILT events (n = 10) limits statistical power, and a clinically meaningful association cannot be excluded. Blood product transfusion guided by viscoelastic haemostatic assessment and rational hemoglobin targets appears to remain an appropriate therapeutic intervention for managing CPB-induced coagulopathy. We found an association between aprotinin use and increased blood product requirement, which warrants further investigation in a larger, dedicated study adjusting for confounders including redo surgery status. These findings provide preliminary reassurance that comprehensive patient blood management approaches may not substantially increase thrombotic complications in this complex patient population. Future research should focus on better characterizing the true mechanistic drivers of ILT—particularly anatomical factors and postoperative anticoagulation strategies—to develop more effective prevention strategies. Larger multicentre prospective studies with standardized anticoagulation protocols and detailed graft characterization are needed to confirm these findings. Meanwhile, clinicians may continue to apply evidence-based transfusion algorithms and goal-directed haemostatic management in patients undergoing aortic arch surgery.

## Figures and Tables

**Table 1:** Demographic and Operative Characteristics.

Parameter	ILT (n = 10)	No ILT (n = 152)	*p* Value
Age (years)	68.6 ± 9.03	68.47 ± 9.82	0.870
Male gender, n (%)	4 (40.0)	91 (59.9)	0.229
Cardiopulmonary bypass time (min)	252.7 ± 93.0	245.6 ± 60.12	0.645
Aortic cross-clamp time (min)	102.5 ± 95.6	103.4 ± 65.1	0.565
Chest drain output 6 h (mL)	216.5 ± 156.6	269.7 ± 274.9	0.997
Chest drain output 24 h (mL)	635.3 ± 292.1	786.9 ± 557.6	0.438
Chest drain output 48 h (mL)	1217.0 ± 575.6	1147.0 ± 818.6	0.536

**Table 2:** Transfusion requirements by ILT status.

Blood Product	ILT (n = 10) Median (IQR)	No ILT (n = 152) Median (IQR)	*p* Value
Red Blood Cells (units)	4.0 (2.0–7.5)	3.0 (1.0–5.0)	0.214
Fresh Frozen Plasma (units)	3.0 (1.5–6.0)	3.0 (1.0–5.0)	0.797
Platelets (units)	2.0 (1.0–2.5)	2.0 (1.0–3.0)	0.761
Cryoprecipitate (units)	1.0 (0.0–2.0)	1.0 (0.0–2.0)	0.437
Fibrinogen Concentrate (grams)	0.0 (0.0–3.0)	0.0 (0.0–0.0)	0.139
Total Blood Products (units) *	12.5 (7.0–18.0)	10.0 (6.0–15.0)	0.897

* Total blood products exclude fibrinogen concentrate (reported in grams rather than units).

**Table 3:** Transfusion volumes by antifibrinolytic agent.

Blood Product	Aprotinin (n = 46) Median (IQR)	TXA (n = 114) Median (IQR)	*p* Value
Red Blood Cells (units)	4.0 (2.0–6.0)	3.0 (1.0–5.0)	0.130
Fresh Frozen Plasma (units)	4.0 (2.0–6.0)	3.0 (1.0–4.5)	<0.001
Platelets (units)	2.0 (1.0–3.0)	2.0 (1.0–2.0)	0.028
Cryoprecipitate (units)	1.0 (0.0–2.0)	1.0 (0.0–2.0)	0.836
Fibrinogen Concentrate (grams)	0.0 (0.0–1.0)	0.0 (0.0–0.0)	0.059
Total Blood Products (units) *	12.0 (8.0–17.0)	9.0 (5.0–14.0)	0.003
No. with ILT (%)	1 (2.2%)	9 (7.9%)	0.232

* Total blood products exclude fibrinogen concentrate (reported in grams rather than units).

## Data Availability

Anonymized data can be made available on request subject to our institution’s data sharing policy.
